# Prevalence Estimation Using a Depression Screening Tool in the National Health and Nutrition Examination Survey: Comparison of Different Cutoffs

**DOI:** 10.1002/mpr.70019

**Published:** 2025-04-03

**Authors:** Ali Mertcan Köse, Paul Petzold, Dario Zocholl, Polychronis Kostoulas, Matthias Rose, Felix Fischer

**Affiliations:** ^1^ Department of Computer Programming Istanbul Ticaret University Istanbul Turkey; ^2^ Charité – Universitätsmedizin Berlin Corporate Member of Freie Universität Berlin and Humboldt Universität zu Berlin Medizinische Klinik Mit Schwerpunkt für Psychosomatik Center for Patient‐Centered Outcomes Research Berlin Germany; ^3^ Institute of Medical Biometry Informatics and Epidemiology University Hospital Bonn Bonn Germany; ^4^ Laboratory of Epidemiology & Artificial Intelligence Faculty of Public & One Health University of Thessaly Karditsa Greece; ^5^ German Center for Mental Health (DZPG) Berlin Germany

**Keywords:** Bayesian latent class models, depression prevalence, National Health and Nutrition Examination Survey, PHQ‐9

## Abstract

**Objectives:**

The National Health and Nutrition Examination Survey (NHANES) in the US relies on the depression screening tool PHQ‐9 to assess depressive symptoms in the general population. For prevalence estimation, PHQ‐9s imperfect diagnostic accuracy can be modeled with a Bayesian Latent Class Model. We investigate the impact of different cutoffs on prevalence estimation.

**Methods:**

We used data from the 16‐th wave of the National Health and Nutrition Examination Survey (NHANES). We assessed the joint posterior distribution to asssess the prevalence of major depression as well as sensitivity and specificity of the PHQ‐9 at cutoffs 5 to 15. We also assessed the impact of weakly and strongly informative prevalence priors.

**Results:**

Data from 9693 participants of the NHANES Wave 2019–2020 were analyzed. Under weakly informative prevalence priors, prevalence estimates ranged from 16.0% (95% CrI: 0.3%–87.8%) when using a cut‐off of 5% to 3.9% (0.2%–12.7%) at 13. More informative prevalence priors led to narrower credible intervals, but the observed data was still in accordance with a wide range of possible MDD prevalence estimates.

**Conclusions:**

Regardless of the cutoff and the prevalence prior chosen, prevalence estimation of major depressive disorders in the NHANES based on the PHQ‐9 is imprecise.

## 1 Introduction

Major depressive disorder (MDD) is one of the most disabling mental disorders worldwide and is characterized by symptoms such as feelings of sadness, exhaustion, and/or guilt, loss of interest, poor concentration and disturbed sleep or appetite. These symptoms substantially influence the perceived health, overall quality of life, social relations, and occupational potential (Iranpour et al. 2022; World Health Organization 2021). Approximately 280 million people worldwide suffer from MDD with a prevalence around 5.0% among adults and 5.8% among people over the age of 60 (World Health Organization 2021). In the United States, 8.4% of adults are estimated to suffer from MDD (National Institute of Mental Health 2022). Hence, MDD is an important public health issue (Weinberger et al. 2018), and accurately estimating its prevalence is vital for informed policy‐making and the development of targeted interventions.

Semi‐structured diagnostic interviews are the reference standard to establish a diagnosis of MDD in research. These interviews require trained diagnosticians with clinical experience to assess symptoms, apply clinical judgment, and rule out alternative explanations for symptoms, such as comorbid conditions. In contrast, fully structured interviews follow an entirely scripted format and can be administered by non‐expert interviewers. While this standardization enhances consistency and efficiency, it may come at the expense of diagnostic accuracy, as there is no opportunity for clinical judgment or the consideration of alternative diagnoses. Such a structured clinical interview is the Mini International Neuropsychiatric Interview (MINI), that is designed for rapid administration but is criticized for being overly inclusive (Levis et al. 2018).

Instead of conducting clinical interviews, researchers commonly revert to self‐report questionnaires to assess symptoms of depression, since these questionnaires allow study participants to report their symptoms independently, without the need for an interviewer. Self‐report measures, typically developed for screening, monitoring, and assessing symptom severity, do not involve clinical evaluation and cannot differentiate between depressive symptoms caused by MDD and those stemming from other medical or psychological conditions. Depression screening tools commonly apply pre‐determined cut‐off thresholds to classify individuals as screening‐positive or screening‐negative for depressive disorder. However, a positive screening result does not confirm a diagnosis and further clinical assessment is required to determine whether depression disorder is present or not (Levis et al. 2019). One of the most widely used screening tools is the Patient Health Questionnaire‐9 PHQ‐9 (Thombs et al. 2018; Wu et al. 2020). A comprehensive individual participant data meta‐analysis of diagnostic studies identified a cutoff score of 10 as optimal, yielding a sensitivity of 85% (95%‐CI: 79%–89%) and a specificity of 85% (95%‐CI: 82%–87%) respectively (Negeri et al. 2021).

It is common practice to report the share of participants that score above a cutoff (usually a score of 10 or above is chosen in the PHQ‐8 and PHQ‐9) as depression prevalence, for example to investigate time trends and predictors of depression prevalence (Wu et al. 2020). However, this approach can substantially overestimate the true prevalence by a factor of two to three, particularly in low‐prevalence populations where false positive test results are common (Fischer et al. 2023; Levis et al. 2020). First, self‐reported symptom measures do not account for clinical significance or functional impairment and cannot distinguish between symptoms attributable to major depressive disorder (MDD) and those arising from other medical or psychological conditions. Hence, high scores are not equivalent to the presence of a specific disorder. Second, screening thresholds are typically designed to maximize case identification rather than to establish a definitive diagnosis, leading to a high proportion of false positives. Hence, screening tools exhibit imperfect diagnostic accuracy, and failure to adjust for their sensitivity and specificity can introduce substantial bias into prevalence estimates.

To address these issues and enable correct prevalence estimation, statistical methods that can account for measurement error, such as Bayesian Latent Class Models should be employed (Joseph et al. 1995; Speybroeck et al. 2013). These Bayesian approaches offer the opportunity to include available evidence on diagnostic accuracy by using sensible prior distributions (Taub et al. 2005) and estimate prevalence even when study‐specific sensitivity and specificity are unknown (McInturff et al. 2004).

Fischer et al. (2023) estimated major depression prevalence across different European countries based on the PHQ‐8 using data from the European Health Interview Survey (EHIS) by incorporating data on the diagnostic accuracy of the PHQ‐8 from an individual participant data meta‐analysis of 27 studies with *n* = 6362 participants Wu et al. (2020) within a Bayesian framework (Bayesian Latent Class Model) (Fischer et al. 2023; Wu et al. 2020). Here, a cutoff of 10 was chosen to distinguish between positive and negative tests and the meta‐analytically derived diagnostic accuracy was provided as suitable prior information in the Bayesian Latent Class Model. Resulting prevalence estimates were broad and despite large differences in the observed number of positive tests, no prevalence differences could be established across European countries.

The objective of this study was therefore to improve methods to account for imperfect diagnostic accuracy that can be applied to all kinds of studies addressing prevalence (e.g., cross‐sectional and longitudinal studies, meta‐analyses). Specifically, we wanted to assess how the chosen cutoff of a depression screening tool affects prevalence estimates and their credible intervals based on number of positive screeners in population‐based studies. We applied Bayesian latent class models to the Depression Screening data collected in the National Health and Nutrition‐Examination Survey (NHANES) in order to obtain prevalence estimates for major depressive disorder in the US general population corrected for imperfect diagnostic accuracy of PHQ‐9 using a wide range of potential cutoffs.

## 2 Materials and Method

### 2.1 Setting and Participants

The National Health and Nutrition Examination Survey (NHANES) is a nationwide survey in the United States, with the aim of assessing the health and nutritional status of Americans by combining interviews with physical examinations and laboratory tests. Since 1999, NHANES has been conducted as a continuous survey by the National Center for Health Statistics (NCHS) (Shim et al. 2011; Stierman et al. 2021).

In this study we used data from the NHANES 2019–2020 survey cycle. Due to the COVID‐19 pandemic, NHANES operations were suspended in mid‐2020 after data collection was completed in just 18 Primary Sampling Units (PSUs). As a result, data collection for the remaining 12 PSUs was canceled, leading to an incomplete and not representative sample of the 2019 ‐ March 2020 data. Hence, unbiased estimates could not be obtained from this partial cycle alone. In order to provide nationally representative estimates, the 2019‐March 2020 data was combined with previously released 2017–2018 data and underwent additional weighting procedures to create the NHANES 2017‐March 2020 pre‐pandemic dataset (Stierman et al. 2021).

### 2.2 Measure

The 9‐item Patient Health Questionnaire (PHQ‐9) is one of the most frequently used screening questionnaires for depression in primary care (Spitzer et al. 1999), and is recommended by the National Quality Forum as both a clinical outcome and performance measure (Zimmerman 2019). As a severity measure, PHQ‐9 scores range from 0 to 27, since each of the 9 items is scored from 0 (not at all) to 3 (nearly every day).

The diagnostic accuracy of PHQ‐9 was investigated in a comprehensive individual participant data meta analysis of 42 studies and 44,503 participants (Negeri et al. 2021). Sensitivity and specificity across potential cutoffs between 5 and 15 points have been modeled in relation to the diagnostic reference standard of semi‐structured interviews (mostly Structured Clinical Interview for DSM SCID) using a bivariate random effects meta‐analytic model. A cutoff score of 10 maximized combined sensitivity and specificity for semi structured interviews (sensitivity = 0.85, 95% CI = 0.79–0.89; specificity = 0.85, 95% CI = 0.82–0.87) (Negeri et al. 2021).

### 2.3 Statistical Analysis

To investigate the performance of different PHQ‐9 cutoffs to estimate the prevalence of major depression, we employed Bayesian Latent Class Models, for each cutoff separately, in Stan (Carpenter et al. 2017). Major depression status was modeled as two unobserved (latent) classes (MDD present/not present), taking into account both the test characteristics of the PHQ‐9 and the observed PHQ‐9 status. Prior information on sensitivity and specificity was incorporated probabilistically as multivariate normal distributions derived froman individual‐participant data meta‐analysis (Negeri et al. 2021) of the diagnostic accuracy of the PHQ‐9. The model was fitted using Markov Chain Monte Carlo Sampling and we report point estimates and 95% credible intervals (Crl) for sensitivity, specificity, and prevalence across the PHQ‐9 cutoff values of 5–15.

For all analyses, we used R (R version 4.2.2) and the packages “nhanesA,” “SASxport,” “rstan,” “ggplot2,” “rcompanion,” “FSA” and “MASS.”

#### 2.3.1 Description of Model

We modeled the proportion of observed positive test results with a Bayesian Latent Class Modeling approach for each PHQ‐9 cutoff from 5 to 15. Our models estimate three cutoff‐specific parameters: sensitivity, specificity and prevalence. We assume that prevalence follows a beta distribution, whereas for logit‐sensitivity and logit‐specificity we assumed a multivariate normal distribution. For each cutoff score *i*, we assumed that the number of individuals who tested positive for depression, denotet as *y*_*i*_, out of the total number of tested individuals, denoted as *n*_*i*_, followed a binomial distribution with a probability of positive test specific to that score, denoted as *p*_*i*_:

$$y_{i}\;∼\;\text{Binomial}\;\left(n_{i},p_{i}\right)$$

where *p*_*i*_ is the sum of the probabilities for true positive (TP_*i*_) and false positive tests (FP_*i*_). These can be expressed in terms of prevalence (Prev_*i*_), sensitivity (*Se*_*i*_) and specificity (Sp_*i*_):

$$p_{i}={\text{TP}}_{i}+{\text{FP}}_{i}={\text{Se}}_{i}*{\text{Prev}}_{i}+\left(1-{\text{Sp}}_{i}\right)*\left(1-{\text{Prev}}_{i}\right)$$

We used a joint multivariate normal prior on the logit of sensitivity and specificity for each cutoff, where the logits are assumed to be normally distributed around a mean logit‐sensitivity (*β*_0*i*_) and mean‐logit specificity (*β*_1*i*_) with a covariance matrix Σ_i_ containing between‐study variances *τ*_1i_ and *τ*_2i_, and between‐study correlation $\rho_{i}$ (Riley et al. 2015).

$$\text{logit}\left(\begin{array}{c} {\text{Se}}_{i}\\ {\text{Sp}}_{i} \end{array}\right)∼N\left[\left(\begin{array}{c} \beta_{1i}\\ \beta_{0i} \end{array}\right),\Sigma_{i}\right],\Sigma_{i}=\left(\begin{array}{cc} \tau_{1i}^{2} & \tau_{1i}\tau_{0i}\rho_{i}\\ \tau_{1i}\tau_{0i}\rho_{i} & \tau_{0i}^{2} \end{array}\right)$$

and a beta prior for Prev,

$$\text{Pre}v_{i}∼\text{Beta}\;\left(a,b\right)$$

#### 2.3.2 Prevalence Priors

Selection of priors is crucial in Bayesian analysis. For the prevalence, we used three different priors:

1. A weakly informative uniform prior Beta (1, 1), which assigns equal probability across all prevalence levels. This mimicks a frequentist approach, where no information on prevalence is included in the model.
2. A prior reflecting vague information on depression prevalence, with 95% of the probability density below 20% and a median prevalence of 5%.
3. A prior reflecting specific information on depression prevalence, with 95% of the probability density below 10% and a median prevalence of 5%.

The respective Beta distributions were constructed using the PriorGen R package (Kostoulas 2018).

#### 2.3.3 Prior of Sensitivity and Specificity

**FIGURE 1:**
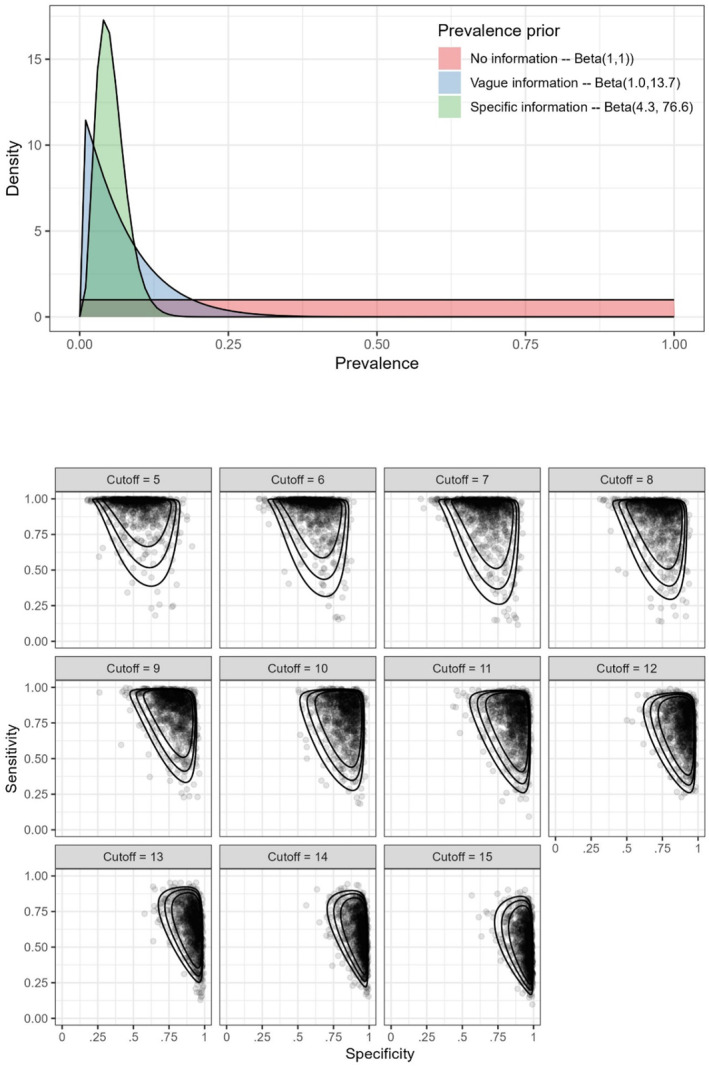
Priors on prevalence, sensitivity and specificity.

In a recent comprehensive individual participant data meta‐analysis (IPD‐MA), a bivariate random‐effects model was used to model diagnostic accuracy of the PHQ‐9 (Negeri et al. 2021). This model estimates sensitivity and specificity simultaneously for a given selected threshold (Simoneau et al. 2017). Prior information about sensitivity (Se_*i*_) and specificity (Sp_*i*_) of the PHQ‐9 was derived from estimates of mean logit‐sensitivity and specificity (*β*_0_ and *β*_1_), between‐study variances *τ*_1_^2^, *τ*_0_^2^, and between‐study correlation *ρ* (Negeri et al. 2021; Riley et al. 2015; Simoneau et al. 2017). For all cutoff values, the prior information that was used is given in Table 1. Figure 1 shows the prior distributions for prevalence as well as the joint prior distribution for sensitivity and specificity. It is evident that low cutoffs result in high sensitivity and low specificity, whereas higher cutoffs result in lower sensitivity but higher specificity.

**TABLE 1:** Parameters used in prior distribution for each cut‐off as well as resulting 95% density regions for sensitivity and specificity.

PHQ‐9 cut‐off (*i*)	*y*	*β*_0_	*β*_1_	*τ*_0_	*τ*_1_	*ρ*	Sensitivity (mean and 95%‐density region)	Specificity (mean and 95%‐density region)
5	2192	3.82	0.13	1.75	0.58	−0.27	0.94 (0.61–1.00)	0.54 (0.29–0.78)
6	1783	3.43	0.45	1.72	0.55	−0.28	0.93 (0.62–1.00)	0.60 (0.35–0.82)
7	1457	3.04	0.74	1.67	0.58	−0.28	0.89 (0.44–1.00)	0.66 (0.42–0.87)
8	1205	2.50	1.03	1.38	0.58	−0.28	0.87 (0.45–0.99)	0.73 (0.50–0.90)
9	972	2.04	1.35	1.12	0.59	−0.36	0.84 (0.47–0.99)	0.78 (0.54–0.93)
10	788	1.73	1.70	1.10	0.67	−0.20	0.81 (0.40–0.98)	0.83 (0.59–0.96)
11	631	1.45	1.99	1.02	0.73	−0.29	0.77 (0.38–0.97)	0.86 (0.64–0.96)
12	507	1.09	2.24	0.87	0.72	−0.27	0.72 (0.34–0.94)	0.89 (0.70–0.97)
13	413	0.70	2.51	0.73	0.73	−0.38	0.67 (0.34–0.90)	0.91 (0.76–0.98)
14	349	0.45	2.80	0.70	0.79	−0.44	0.61 (0.28–0.87)	0.92 (0.77–0.99)
15	279	0.09	3.11	0.69	0.87	−0.38	0.52 (0.21–0.82)	0.94 (0.82–0.99)

#### 2.3.4 Model Fitting

All models were fitted in Stan using Markov Chain Monte Carlo Sampling (4 chains, 5000 iterations, 2500 warm‐up iterations): we examined trace plots, R‐hat values, effective sample size and autocorrelation plots to assess model convergence.

#### 2.3.5 Interpretation

We assessed the joint posterior distribution of the model parameters Prev_*i*_, Se_*i*_, Sp_*i*_. We reported posterior median and 95% credible intervals (Crl) of Prev_*i*_, Se_*i*_, and Sp_*i*_ for each cutoff value and compared the marginal and joint posterior distributions of Se_*i*_ and Sp_*i*_ to their respective prior distributions.

### 2.4 Web Application

In order to enable reproduction of our analysis and to estimate depression prevalence taking into account the imperfect diagnostic accuracy of the PHQ‐9, we provide a web application (http://www.common‐metrics.org/MDD_prevalence_estimation.php), where researchers can apply the model and priors used in this study to their own data.

## 3 Results

Overall, we used data from 9693 participants aged 18 years and above. Variables of participant characteristics include age, gender, education, marital status, occupation, family monthly poverty level category and weight. Participant characteristics variables are presented in Table 2. We excluded 1392 participants from the analyses due to missing item responses in the PHQ‐9, leaving 8301 participants for prevalence estimation.

**TABLE 2:** Participant characteristics (overall *n*, age, sex, occupation, family status).

Variables		Frequency	Percent
Gender	Male	4718	48.67
	Female	4975	51.33
	Total	9693	100
Education	Less than 9th grade	719	7.42
	9–11th grade (includes 12th grade with no diploma)	1041	10.74
	High school graduate/GED or equivalent	2225	22.95
	Some college or AA degree	2975	30.69
	College graduate or above	2257	23.28
	Refused	2	0.02
	Don't know	13	0.13
	Missing	461	4.76
	Total	9693	100
Marital status	Married/living with partner	5279	54.46
	Widowed/divorced/separated	2148	22.16
	Never married	1795	18.52
	Refused	8	0.08
	Don't know	2	0.02
	Missing	461	4.76
	Total	9693	100
Occupation	Working at a job or business	5241	54.07
	With a job or business but not at work	212	2.19
	Looking for work. or	392	4.04
	Not working at a job or business?	3844	39.66
	Refused	1	0.01
	Don't know	3	0.03
	Total	9693	100
Family monthly poverty level category	Monthly poverty level index = 1.30	2732	28.2
	1.30 < monthly poverty level index = 1.85	1288	13.3
	Monthly poverty level index > 1.85	4407	45.5
	Refused	69	0.7
	Don't know	296	3.1
	Missing	901	9.3
	Total	9693	100

*Note:* Age: 49.59 ± 18.61; Weight: 83.33 ± 23.22.

Model sampling performed well without any indication of problems. Examination of trace plots, autocorrelation plots, R‐hat values (all parameters < 1.02) and effective sample size (all parameters > 800) indicated appropriate exploration of the posterior distribution.

**FIGURE 2:**
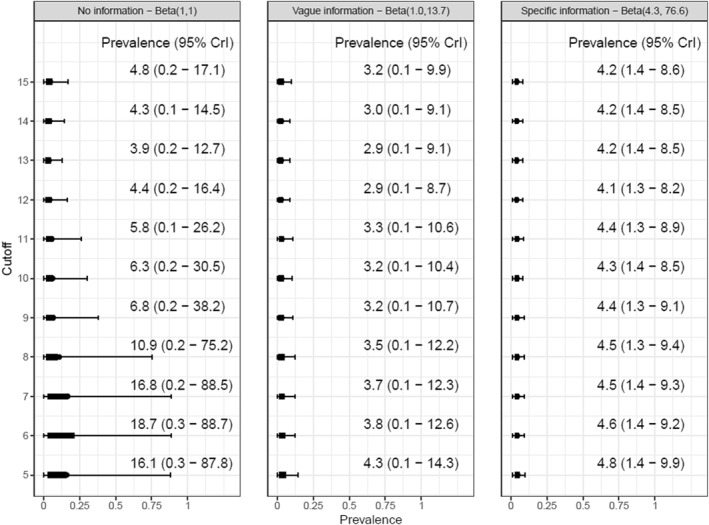
Prevalence estimates based on different cutoffs and with different prevalence priors.

Estimates of prevalence for each cutoff and the three different prevalence prior distributions are presented in Figure 2. Overall, we see that higher cutoffs yield smaller prevalence estimates, and that there is a substantial influence of the prevalence prior on the size and precision of the prevalence posterior. As expected, using a uniform prevalence prior yields the most imprecise prevalence estimates, but the credibility intervals for prevalence estimates remain wide for the more informative priors (vague and specific information).

**FIGURE 3:**
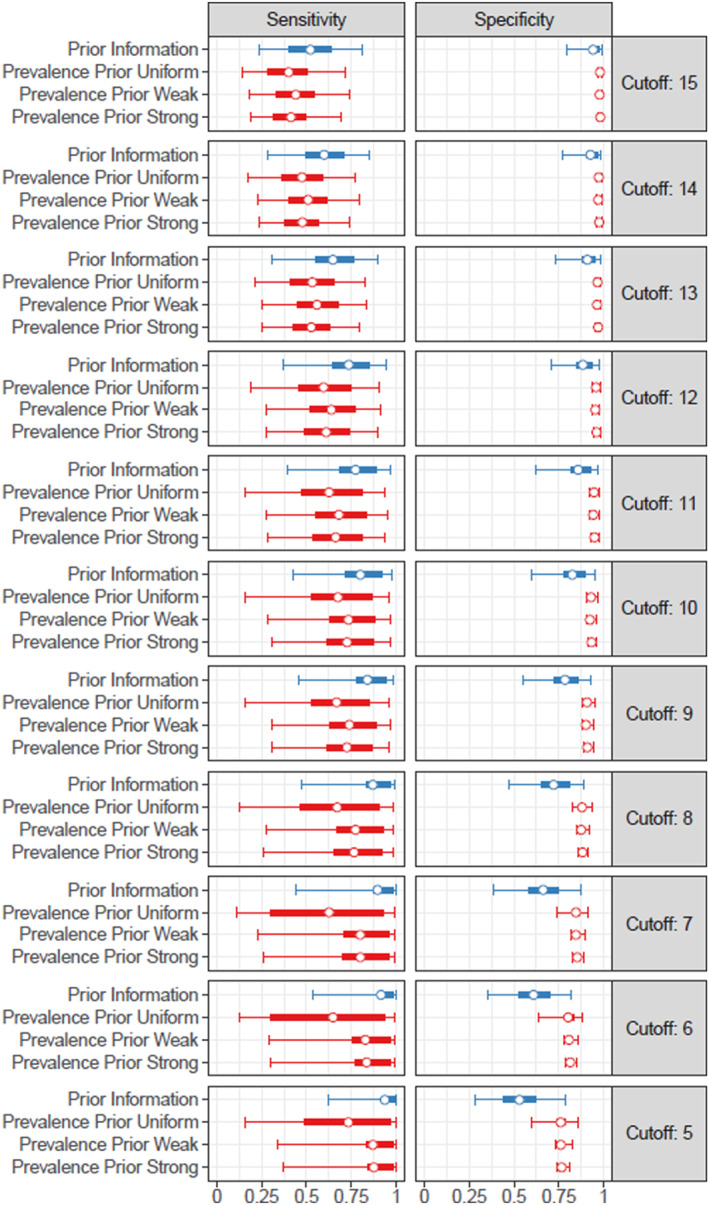
Sensitivity and specificity estimates based on different cutoffs and with different prevalence priors.

Figure 3 shows the priors and the posterior distribution for sensitivity and specificity. For specificity, it is apparent that the posterior distribution is considerably narrower than the prior information suggests. On the contrary, the posterior distribution of sensitivity is slightly broader than prior. This pattern is consistent over all cutoffs. Again, when utilizing a uniform distribution as prevalence prior, we yield most imprecise results.

## 4 Discussion

We conducted this study to investigate the impact of using different cutoffs on prevalence estimation using a depression screening tool with imperfect diagnostic accuracy in a large population based survey. For this purpose, we estimated the prevalence of major depression disorder based on different PHQ‐9 cutoffs by using Bayesian latent class analysis incorporating prior information on sensitivity and specificity.

Our findings highlight key limitations of the common practice of estimating depression prevalence based on a single cutoff from the PHQ‐9. First, prevalence estimates varied substantially depending on the cutoff used. For example, using a cutoff of 5 resulted in an estimated prevalence of 16.1%, whereas a cutoff of 15 yielded a much lower estimate of 4.8%. This discrepancy arises due to differences in sensitivity and specificity across cutoffs. Second, prior information on diagnostic accuracy plays a crucial role in shaping prevalence estimates. While incorporating specificity estimates from a large‐scale diagnostic accuracy meta‐analysis was intended to improve precision, we found that these priors were inconsistent with the NHANES data. This inconsistency suggests that standard diagnostic accuracy estimates may not generalize well to population‐based settings. Third, even with informative priors, our approach could not generate precise prevalence estimates. While our analysis using vague prevalence priors suggested a central prevalence estimate of around 4%, the credible intervals remained wide (ranging from 0% to 15%), reflecting substantial uncertainty. Higher cutoffs generally produced narrower intervals, but the estimates were still too imprecise for confident inference.

Overall, these findings emphasize a critical issue with the naïve approach of ignoring diagnostic imperfectness: simply reporting the proportions of individuals above a cutoff ignores the high rate of false positives, particularly in low‐prevalence populations. This leads to exaggerated prevalence rates, potentially misguiding researchers and policymakers.

Under the most informative prevalence prior and a cutoff of 12, we estimated the prevalence of depression among U.S. adults to be between 1.3% and 8.2% with the median estimate being 4.1%. Previous studies from NHANES reported higher prevalence rates such as 8.0% (95% CI: 7.3%–8.7%) among U.S. adults from 2015 to 2018 (Cao et al. 2020) and 7.5% prevalence from 2005 to 2016 (Iranpour et al. 2022), which indicates the possibility of an overestimation of prevalence values when imperfect diagnostic accuracy is not considered. Furthermore, our credible intervals indicate substantially larger uncertainty about the actual prevalence.

Consistent with prior research using population‐based surveys in Europe (Fischer et al. 2023), we found that prior information from diagnostic studies (Negeri et al. 2021) did not align well with observed data in population‐based surveys. This discrepancy was particularly notable in specificity, where the number of positive tests was lower than expected based on previous specificity estimates. Consequently, we estimated the specificity in the NHANES data to be higher than previously thought. In contrast, the NHANES data provided little information on PHQ‐9 sensitivity, leading to even broader posterior distributions. The mismatch likely arises because diagnostic studies are often conducted in populations at higher risk for MDD than the general population. Overall, primary diagnostic studies have shown that the diagnostic accuracy of the PHQ‐9 varies significantly across studies, leading to broad priors on its diagnostic accuracy (Levis et al. 2019; Negeri et al. 2021; Wu et al. 2020).

We were unable to establish precise prevalence estimates for depression based solely on the PHQ‐9 under any condition. Achieving such precision would require much more accurate information on diagnostic accuracy of the PHQ‐9 within the specific study population. This could potentially be accomplished through a two‐step design, where structured clinical interviews are conducted in a subsample of participants to gather study‐specific data on diagnostic accuracy. Another promising approach could be to incorporate information from all cutoffs simultaneously to estimate prevalence, rather than relying on a single cutoff. Our results indicate that the information about the prevalence varies across different cutoffs and combining this information into a unified prevalence estimate might improve the accuracy of estimation. Developing appropriate methodological frameworks for this integration remains an open area of research. Further, we based our analysis on the PHQ‐9 sum score to determine depression status, as to date diagnostic accuracy information is available only for this approach. Incorporating item‐level test characteristics could be a potential way to improve prevalence estimation in future research.

### 4.1 Strengths and Limitations

In this study, we used the best available evidence to model the diagnostic accuracy of the PHQ‐9 in a population based survey. The data analyzed was sourced from the NHANES dataset, which was collected prior to the COVID‐19 pandemic, and results may therefore not fully reflect current prevalence rates. Hence, the prevalence estimates obtained should be interpreted with caution. Additionally, nonresponse to the PHQ‐9 could introduce biases into the dataset, potentially leading to self‐selection bias, where individuals with more severe depressive symptoms are less likely to complete the survey. Further, our study relied on the results of previous studies on the diagnostic accuracy of the PHQ‐9 that mainly included at‐risk populations, which might not reflect the (current) diagnostic accuracy among the general population. Ideally, a two‐step approach to obtain information on the diagnostic accuracy in the specific population should be employed, where clinical interviews are administered to a sub‐sample of the desired population. Thereby, we used a multivariate normal prior distribution based on the model parameters reported by the diagnostic accuracy individual participant data meta‐analysis. Although this is one of the most widely used modeling techniques for diagnostics accuracy, there might be alternative prior definitions, for example accounting for violations of multivariate normality. Also, prior distributions on prevalence were not developed using actual information on depression prevalence in the US, but rather depict approximations of potential knowledge on depression prevalence.

## 5 Conclusion

In this study, we investigated how the choice of different cutoffs affects Bayesian Latent Class Models and the resulting estimates of prevalence and diagnostic accuracy in population‐based studies. Our findings indicate that, regardless of the cutoff used, prevalence estimates based on the PHQ‐9, a widely used depression screener, remain imprecise. To obtain more precise prevalence estimates, collecting sample‐specific information on diagnostic accuracy is essential, for example, through a two‐step design in which a subset of participants is also assessed using a validated diagnostic tool.

## Author Contributions

**Ali Mertcan Köse:** conceptualization, methodology, software, data curation, formal analysis, writing – original draft. **Paul Petzold:** methodology, software, formal analysis, writing – review and editing. **Dario Zocholl:** conceptualization, methodology, writing – review and editing. **Polychronis Kostoulas:** conceptualization, methodology, writing – review and editing. **Matthias Rose:** resources, writing – review and editing. **Felix Fischer:** project administration, conceptualization, methodology, software, data curation, formal analysis, supervision, writing – review and editing.

## Ethics Statement

This study is analyzed from secondary data on NHANES website, thereby no further ethics approval for conducting the present study is required.

## Conflicts of Interest

The authors declare no conflicts of interest.

## Data Availability

The data that support the findings of the study are available on https://wwwn.cdc.gov/nchs/nhanes/.
